# Identification of an energy metabolism-related signature associated with clinical prognosis in diffuse glioma

**DOI:** 10.18632/aging.101625

**Published:** 2018-11-08

**Authors:** Zhengui Zhou, Ruoyu Huang, Ruichao Chai, Xiaohong Zhou, Zhiping Hu, Wenbiao Wang, Baoguo Chen, Lintao Deng, Yuqing Liu, Fan Wu

**Affiliations:** 1 Department of Molecular Neuropathology, Beijing Neurosurgical Institute, Capital Medical University. Beijing 100050, China; 2 Department of Cerebral Surgery, The People’s Hospital of Gongan County, Hu Bei, Gongan 434300, China; 3 Department of Neurosurgery, Beijing Tiantan Hospital, Capital Medical University, Beijing 100050, China; 4 Chinese Glioma Genome Atlas Network (CGGA) and Asian Glioma Genome Atlas Network (AGGA), Beijing 100050, China; * Equal contribution

**Keywords:** glioma, energy metabolism, signature, prognosis

## Abstract

Now, numerous exciting findings have been yielded in the field of energy metabolism within glioma cells. In addition to aerobic glycolysis, multiple catabolic pathways are employed for energy production. However, the prognostic significance of energy metabolism in glioma remains obscure. Here, we explored the relationship between energy metabolism gene profile and outcome of diffuse glioma patients using The Cancer Genome Altas (TCGA) and Chinese Glioma Genome Altas (CGGA) datasets. Based on the gene expression profile, consensus clustering identified two robust clusters of glioma patients with distinguished prognostic and molecular features. With the Cox proportional hazards model with elastic net penalty, an energy metabolism-related signature was built to evaluate patients’ prognosis. Kaplan-Meier analysis found that the acquired signature could differentiate the outcome of low and high-risk groups of patients in both cohorts. Moreover, the signature, significantly associated with the clinical and molecular features, could serve as an independent prognostic factor for glioma patients. Gene Ontology (GO) and Gene Set Enrichment Analysis (GSEA) showed that gene sets correlated with high-risk group were involved in immune and inflammatory response, with the low-risk group were mainly related to glutamate receptor signaling pathway. Our results provided new insight into energy metabolism role in diffuse glioma.

## Introduction

Energy metabolic reprogramming has been a hallmark of cancer cells, which enable tumor cells to generate ATP for maintaining the reduction-oxidation balance and macromolecular biosynthesis—processes that are required for cell growth, proliferation and migration [1]. Many cancers have long been thought to limit their energy metabolism largely to glycolysis producing large amounts of lactate even in the presence of oxygen, a phenomenon known as the Warburg Effect [2]. In comparison to normal cells, tumor cells prefer to incomplete, non-oxidative metabolism of glucose. Until now, it is widely accepted that glucose is the main energy source of cancer cells. However, awareness that the metabolic phenotype of cancer cells is heterogeneous is growing. Some tumor cells are predominantly glycolytic, whereas others with the given tumor have an oxidative phosphorylation (OXPHOS) metabolic phenotype [3,4]. Increasing evidences show that there is a metabolic symbiosis between glycolytic and oxidative tumor cells. For example, Lactate and pyruvate generated by glycolysis can be transferred to and used as substrates for tricarboxylic acid (TCA) intermediates and ATP production by the neighbor cancer cells [5]. Similarly, malignant tumor cells also can take up free fatty acids and ketones released by adjacent catabolic cells, which will fuel the mitochondrial OXPHOS for energy production [6,7]. In addition, it has been reported that glutamine can also be metabolized by TCA cycle to produce energy [8]. Under hypoxic condition, experiments showed that glutamine-driven mitochondrial OXPHOS accounts for most of ATP production [9]. A deeper understanding of Energy metabolism in tumors could offer a vital step forward in the development of new treatments.

Glioma is the most common form of primary malignant brain tumor, with an incidence of 5-6 cases per 100000 persons per year. Glioblastoma (GBM), a highly aggressive tumor, approximately accounts for 55% of glioma with a dismal median survival of 14-16 months [10]. In addition to the diffuse and infiltrative nature, GBM show strong heterogeneity between patients as well as within individual tumor, which leads to the resistance and inevitable recurrence [11,12]. Despite aggressive treatments, such as surgical resection followed by radiotherapy and chemotherapy, the outcomes of patients with GBM remain very poor [13]. There is an urgent need to find new therapies to improve prognosis for these patients. Accumulating studies have shown that multiple catabolic pathways are involved in energy metabolism of glioma cells [14]. Lin et al reported that primary glioblastoma cells were highly oxidative and largely unaffected by treatment with glycolysis inhibitors, indicating the co-existence of glycolysis and OXPHOS [15]. In particular, glioma stem cells exhibit less glycolytic phenotype compared with their differentiated progeny [16]. Increasingly, recent evidences have shown that glioma cells can also use fatty acids as a substrate for energy production. Inhibition of fatty acid beta-oxidation could reduce the proliferation of glioma cells [17]. However, the local energy metabolic status and its prognostic value in patients with glioma are still remaining to be further elucidated.

In this study, we inquired the energy metabolic profile and its clinical value in patients with diffuse glioma using the TCGA and CGGA RNA sequencing data. Based on the gene expression profile, patients could be classified into two robust groups with significant difference in prognosis and molecular features. Then, we developed an energy metabolism-related signature for assessing the prognosis of glioma patients with TCGA dataset, which was further validated in CGGA dataset. This signature was closely associated with patients’ outcome and could serve as an independent pathological factor. To summarize, our results uncovered a strong association between energy metabolism status and clinical prognosis in diffuse glioma.

## RESULTS

### IDH-wt and IDH-mut LGG show distinct expression profile of energy metabolism genes

To profile the energy metabolism status of glioma, a cohort of 550 patients with RNA sequencing data and clinical information was obtained from the TCGA database. Two energy metabolism-related gene sets were downloaded and integrated into one gene set which contained 587 genes. Within this obtained gene set, 41 genes were involved in carbohydrate metabolism, 73 genes in lipid metabolism and 144 genes in protein metabolism (Supplementary Figure 1A-C). SAM and GO analyses found 25 carbohydrate metabolism genes were differentially expressed between IDH-wt and IDH-mut LGG (Supplementary Figure 1D). Most of the increased genes in IDH-mut LGG were involved in chondroitin sulfate biosynthetic process, while IDH-wt LGG exhibited an enrichment of glycosaminoglycan biosynthetic process (Supplementary Figure 1E). For lipid metabolism, 16 upregulated genes in IDH-mut LGG were mainly involved in fatty acid biosynthetic, while 23 increased genes in IDH-wt LGG were related to bile acid biosynthetic and oxidation-reduction process (Supplementary Figure 1F and G). For protein metabolism, IDH-mut LGG displayed enrichment of translational initiation, whereas IDH-wt LGG exhibited enrichment of protein N-linked glycosylation (Supplementary Figure 1H and I). These results suggested a significant difference of energy metabolism status between IDH-wt and IDH-mut LGG.

**Figure 1:**
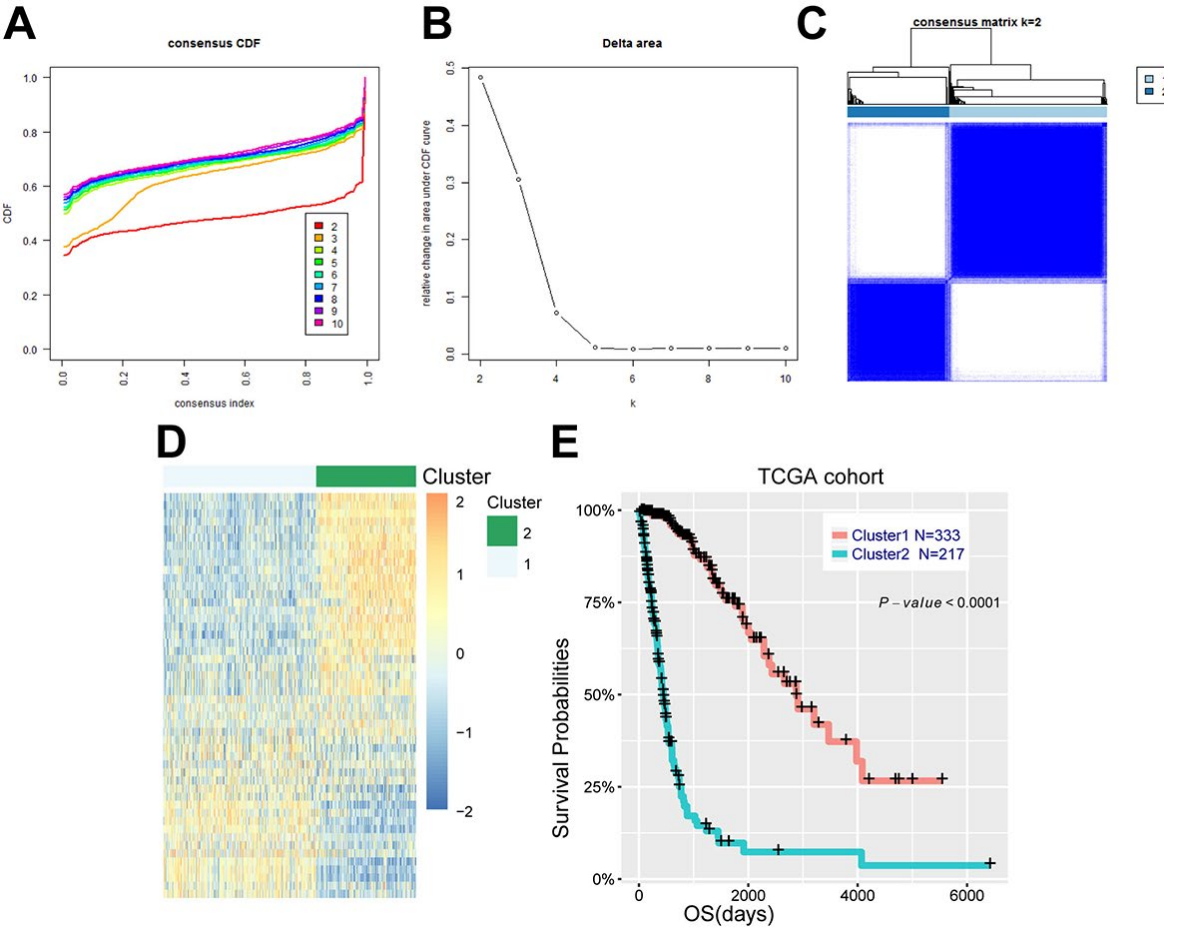
**Energy metabolism-related genes could distinguish diffuse glioma patients with different clinical and molecular features.** (**A**) Consensus clustering CDF for k = 2 to k = 10. (**B**) Relative change in area under CDF curve for k = 2 to k = 10. (**C**) Consensus clustering matrix of 550 samples from TCGA dataset for k = 2. (**D**) Heat map of two clusters defined by the top 50 variable expression genes. (**E**) survival analysis of patients in cluster 1 and cluster 2.

### Identification of an energy metabolism-related prognostic signature in diffuse glioma

We further explored the association between energy metabolism status and outcome of diffuse glioma patients. Consensus clustering found that patients could be classified into two robust groups (Figure 1A-C). Figure 1D showed the heat map of these two clusters defined by the top 50 variable expression genes. Kaplan-Meier analysis revealed that patients in cluster 1 had a significantly longer OS than those in cluster 2 (Figure 1E, *P*<0.001). To further detect the difference between these two clusters of patients, Chi-square test was performed. Patients in cluster 1 were mainly younger, lower grade, proneural or neural subtype, IDH mutational and MGMT promoter methylated (P<0.001), while cluster 2 represented older, high grade, classical or mesenchymal subtype, IDH wild type, and MGMT promoter unmethylated (P<0.001) (Table 1). Similarly, the CGGA cohort of 309 patients with RNA sequencing data and clinical information was also downloaded and analyzed, and consistent results were observed (Supplementary Figure 2, Supplementary Table 1). These results indicated that expression of energy metabolism-related genes was closely correlated with patients’ prognosis and molecular features in diffuse glioma.

**Table 1:** Characteristics of patients in class 1 and class 2 in TCGA cohort.

**Characteristics**	**n**	**Class 1**	**Class 2**	***P*-value**
**Total Cases**	550	333	217	
**Age**				
≤48	287	240	47	**<0.001**
>48	263	93	170	
**Gender**				
Male	319	185	134	0.321
Female	231	148	83	
**Subtype**				
Classical	141	6	135	**<0.001**
Mesenchymal	31	1	30	
Proneural	345	299	46	
Neural	33	27	6	
**Grade**				
II	191	181	10	**<0.001**
III	211	151	60	
IV	148	1	147	
**IDH**				
Mut	338	319	19	**<0.001**
WT	212	14	198	
**MGMT promoter**				
Methylated	383	302	81	**<0.001**
Unmethylated	135	31	104	
NA	32	0	32	

IDH = isocitrate dehydrogenase; MGMT = methylguanine methyltransferase.

**Figure 2:**
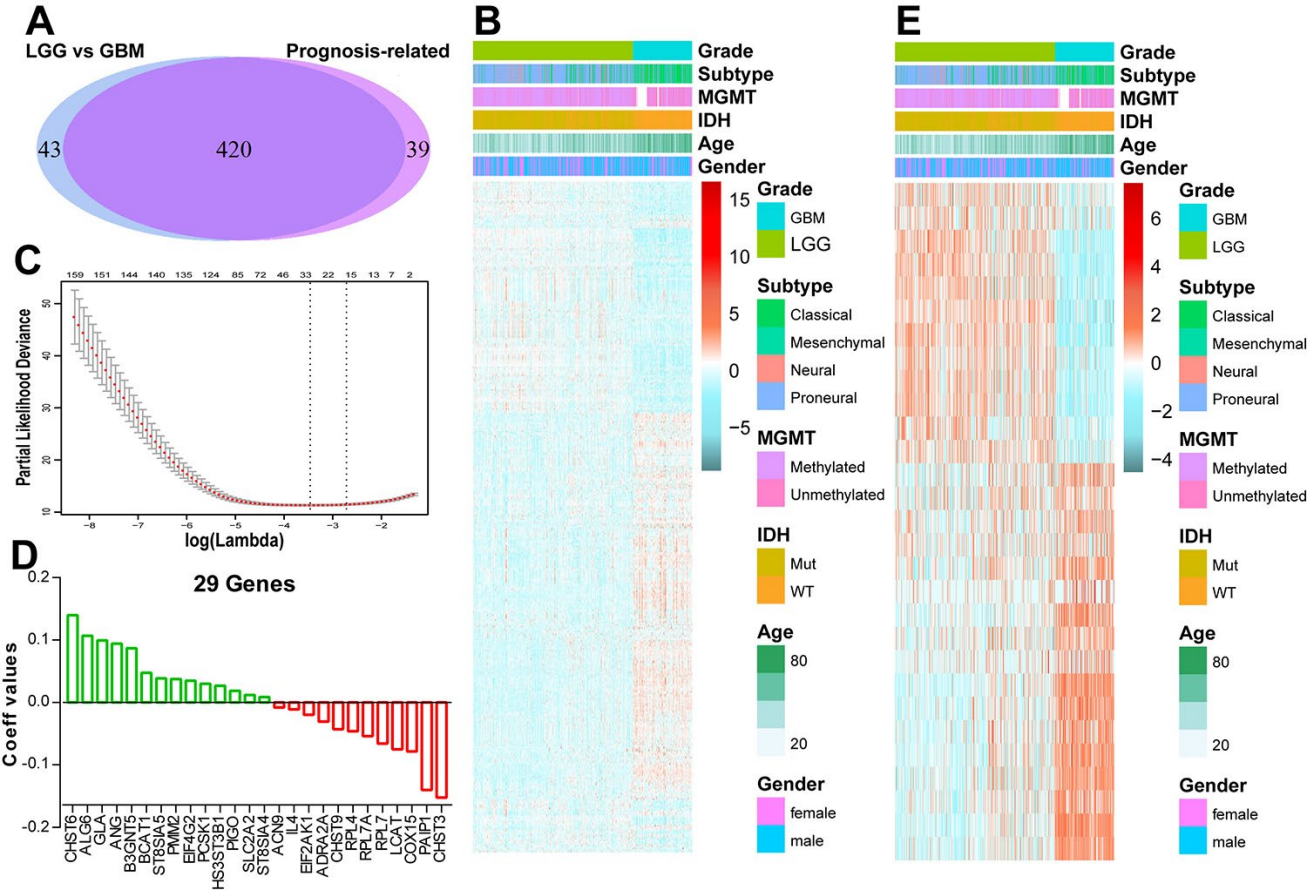
**Identification of an energy metabolism-related signature by Cox proportional hazards model in TCGA cohort.** (**A**) Venn diagram shows prognosis-related genes which are also differentially expressed between LGG and GBM. (**B**) Heat map of 420 energy metabolism-related genes correlated with patients’ OS. (**C**) Cross-validation for tuning parameter selection in the proportional hazards model. (**D**) Coefficient values for each of the 29 selected genes. (**E**) Heatmap of the 29 genes of the signature based on the risk score value.

Considering the strong link between patients’ prognosis and energy metabolism status, we proposed to develop an energy metabolism-related signature for prognosis prediction. SAM analysis found that 463 genes were differentially expressed between LGG and GBM based on the *P* value. Univariate Cox regression analysis revealed 420 out of the differential genes were significantly correlated with patients’ OS, as shown in Figure 2A and B. Then, we applied a Cox proportional hazards model for selecting genes with best prognostic value (Figure 2C). A 29-gene signature was identified (Figure 2D and E) and the risk score was calculated with their expression level and regression coefficients. The biological function of these 29 genes was annotated with GO analysis (Supplementary Figure 3). For the CGGA validation set, the risk scores of patients were computed with the same regression coefficients.

### 29-gene signature shows strong power for prognosis assessment

**Figure 3:**
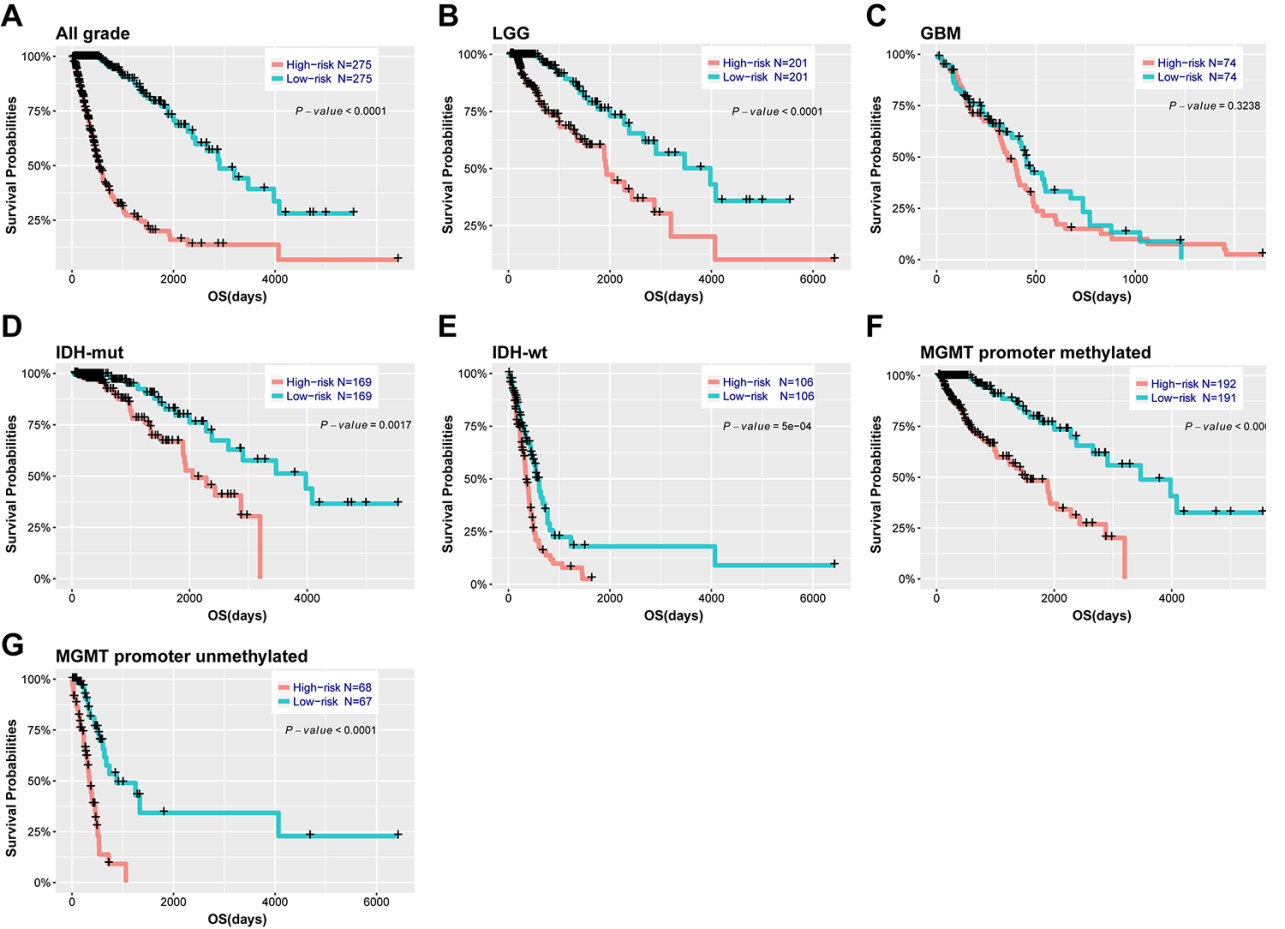
**Outcome prediction of the 29-gene signature in stratified patients of TCGA cohort.** (**A-G**) survival analysis of the signature in patients stratified by grade, IDH and MGMT promoter status.

Based on the median risk score, patients were assigned into high-risk and low-risk groups. Kaplan-Meier analysis showed patients in low-risk group had a significantly longer OS than those in high-risk group (Figure 3A, *P*<0.001). Then, we further explored the prognostic value of this signature in stratified patients by grade, IDH status, MGMT promoter status. The similar results were observed in most stratified patients expect patients with GBM (Figure 3B-G). Similarly, the prognostic value of this signature was also evaluated in the CGGA validation set. Consensus results were obtained by Kaplan-Meier analysis (Supplementary Figure 4). Further stratified analyses also revealed that high risk score conferred reduced OS in molecular subgroups (LGG IDH-wt, LGG IDH-mut and GBM IDH-mut) in both cohorts (Supplementary Figure 5). Univariate and multivariate Cox regression analysis revealed that this risk score was significantly corelated with patients’ OS (95% CI=1.415-2.907, *P*<0.001), independent of age, gender, grade, subtype, IDH and MGMT promoter status (Table 2). Furthermore, the risk score could also serve as an independent prognostic factor in CGGA cohort (95% CI=1.161-2.086, *P*=0.003) (Supplementary Table 2).

**Table 2:** Univariate and multivariate Cox regression analysis of clinical pathologic features for OS in TCGA cohort.

**Characteristics**	**Univariate analysis**				**Multivariate analysis**		
	**HR**	**95% CI**	***P*-value**		**HR**	**95% CI**	***P*-value**
**Age**	1.076	1.063-1.089	**<0.001**		1.059	1.042-1.076	**<0.001**
**Gender**	0.957	0.705-1.299	0.779				
**Grade**	5.285	4.047-6.902	**<0.001**		1.315	0.888-1.946	0.171
**Subtype**	2.398	2.038-2.822	**<0.001**		0.973	0.754-1.255	0.832
**IDH**	0.101	0.07-0.144	**<0.001**		1.517	0.568-4.052	0.405
**MGMT promoter**	0.276	0.196-0.39	**<0.001**		0.812	0.546-1.208	0.305
**Risk score**	2.434	2.144-2.764	**<0.001**		2.028	1.415-2.907	**<0.001**

HR = hazard ratio; CI = confidence interval; IDH = isocitrate dehydrogenase; MGMT = methylguanine methyltransferase.

**Figure 4:**
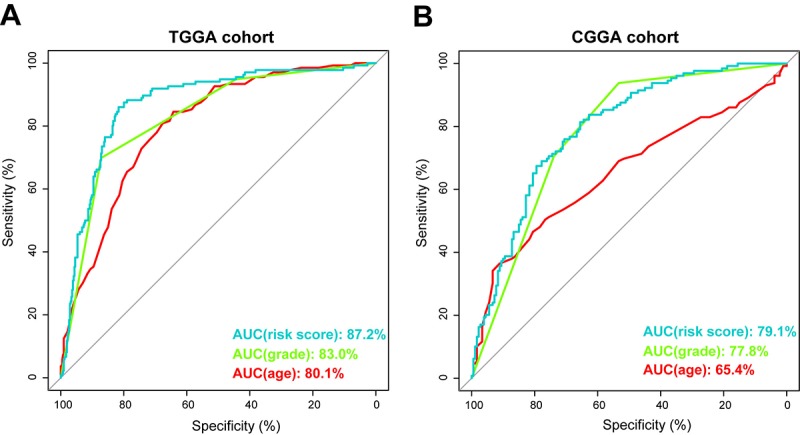
**Prognostic power of the identified 29-gene signature in TCGA and CGGA cohorts.** (**A**) ROC curve analysis of age, grade and risk score in TCGA cohort. (**B**) ROC curve analysis of age, grade and risk score in CGGA cohort. AUC, area under the curve.

Using ROC curve, we further evaluated the predictive accuracy by computing AUC (area under the curve) of risk score, age and grade. The AUC of risk score (87.2%) was much higher than that of age (80.1%) and grade (83.0%) (Figure 4A). Moreover, The AUC of risk score (79.1%) was substantially higher in CGGA validation set (Figure 4B). These data demonstrated the powerful ability of the energy metabolism-related signature for predicting prognosis.

### Energy metabolism-related signature is associated with pathologic features in diffuse glioma

**Figure 5:**
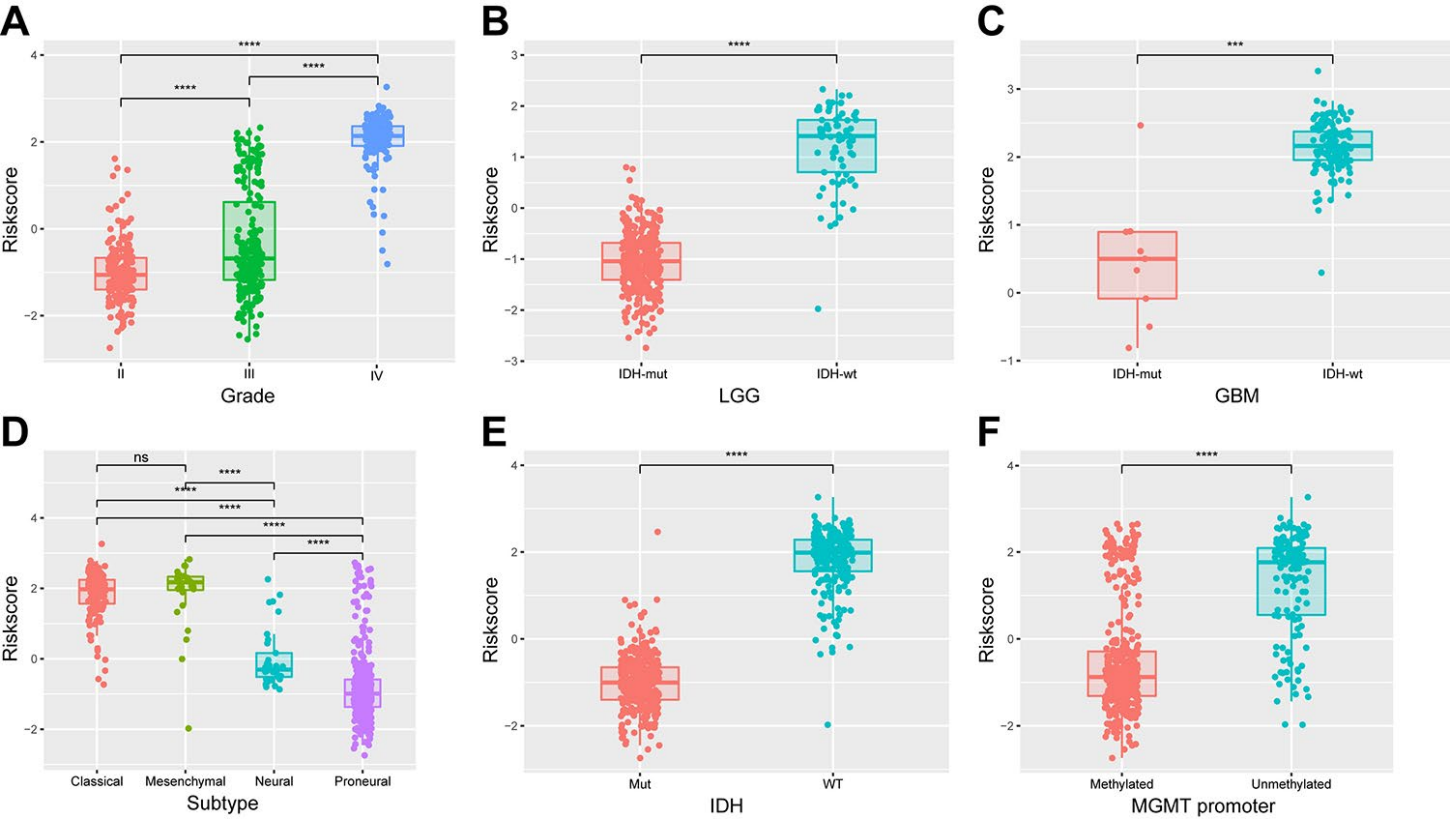
**Association between the energy metabolism-related signature and pathologic features in TCGA cohort.** (**A-F**) Distribution of the risk score in stratified patients by grade, subtype, IDH and MGMT promoter status.

We next determined whether the 29-gene signature was related to patients’ clinical molecular features. Patients were arrayed based on their risk scores. The signature scores distributed differently in stratified patients, with high level in high grade, classic or mesenchymal, IDH wild type and MGMT unmethylated patients (Figure 5). The statistical difference of these features between high and low-risk groups was evaluated using chi-square test. Except gender, most of features were found different between risk groups (Table 3, *P*<0.001). Additionally, similar results were obtained in CGGA cohort of glioma patients (Supplementary Figure 6, Supplementary Table 3). These findings indicated a significant correlation between energy metabolism signature and pathologic features in diffuse glioma.

**Table 3:** Characteristics of patients in low-risk and high-risk groups in TCGA cohort.

**Characteristics**	**n**	**Risk score**		***P*-value**
		**Low**	**High**	
**Total Cases**	550	275	275	
**Age**				
≤48	287	197	90	**<0.001**
>48	263	78	185	
**Gender**				
Male	319	156	163	0.307
Female	231	119	112	
**Subtype**				
Classical	141	2	139	**<0.001**
Mesenchymal	31	1	30	
Proneural	345	264	81	
Neural	33	8	25	
**Grade**				
II	191	155	36	**<0.001**
III	211	119	92	
IV	148	1	147	
**IDH**				
Mut	338	274	64	**<0.001**
WT	212	1	211	
**MGMT promoter**				
Methylated	383	257	126	**<0.001**
Unmethylated	135	18	117	
NA	32	0	32	

IDH = isocitrate dehydrogenase;

MGMT = methylguanine methyltransferase.

### Functional annotation of 29-gene signature

**Figure 6:**
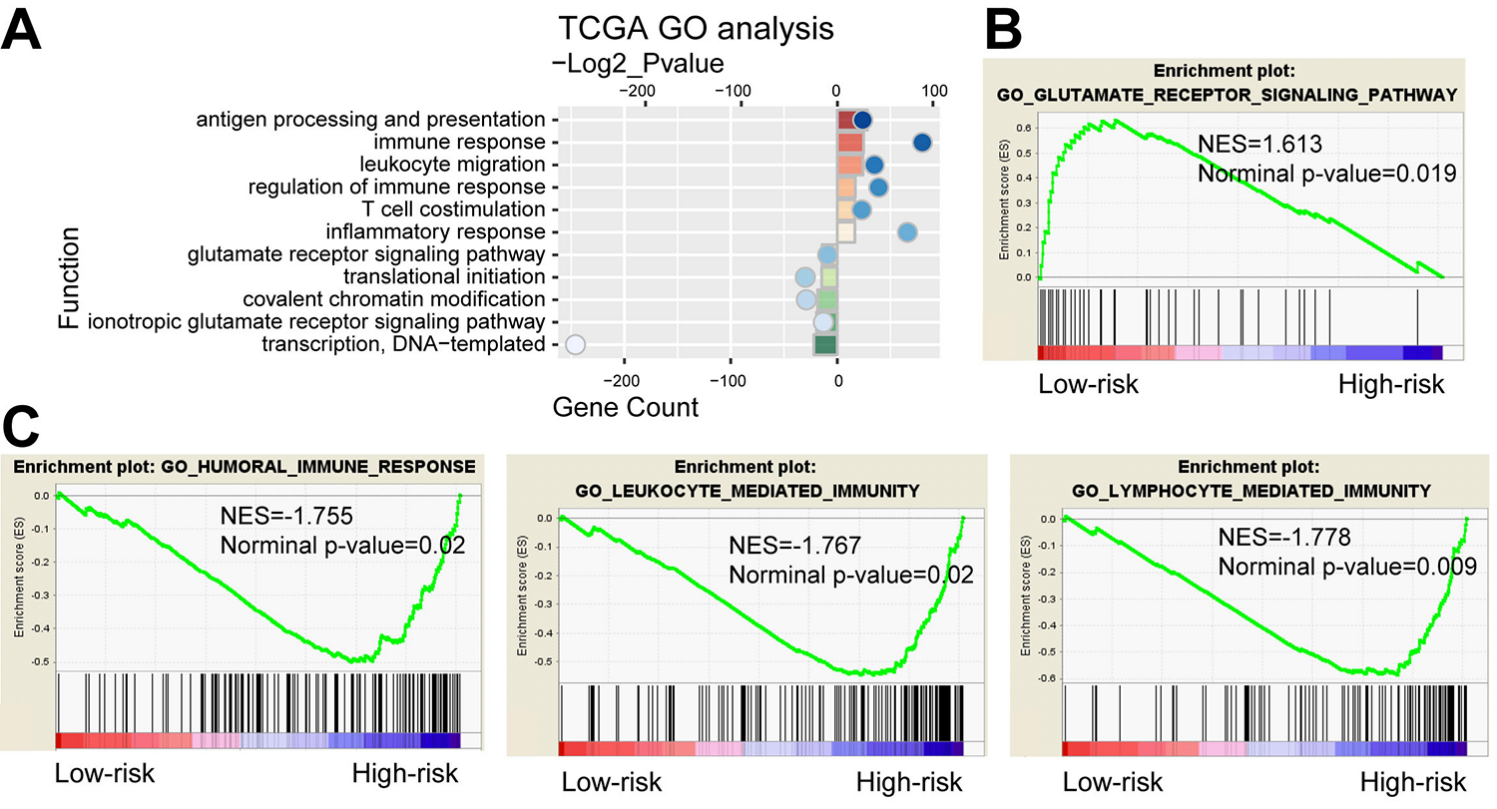
**Functional analysis of the 29-gene signature.** (**A**) GO annotations based on the top 2000 genes positively and negatively associated with the 29-gene signature. (**B-C**) GSEA analysis based on the median value of risk score.

We further compared gene expression between the high-risk and low-risk groups. PCA showed that high and low-risk groups of patients tended to distribute in two sides clearly in both TCGA and CGGA cohort (Supplementary Figure 7). Based on the top 2000 genes of differential expression (*P*<0.05, ranked by fold change) identified by SAM, GO analysis revealed that antigen processing and presentation, immune response, inflammatory response and T cell costimulation were significantly enriched in high-risk group, while the low-risk group showed enrichment of translational initiation and glutamate receptor signaling pathway (Figure 6A). GSEA found that the differentially expressed genes in two groups were associated with humoral immune response, leukocyte mediated immunity, lymphocyte mediated immunity and glutamate receptor signaling pathway (Figure 6B and C). As shown in Supplementary Figure 8, analysis of the CGGA cohort displayed consensus results. Moreover, we also performed functional analyses in LGG and GBM respectively. Consequently, GO and GESA analyses showed similar outcomes (Supplementary Figure 9). The corresponding biologic functions might contribute to patients’ high risk and poor prognosis.

## DISCUSSION

Increasing evidence has revealed that metabolism deregulation is one of the emerging hallmarks of cancer cells. Energy metabolic difference between normal and tumor cells has attracted extensive attention worldwide for decades. In glioma, resent studies demonstrated that multiple catabolic pathways are involved in its energy metabolism, such as glycolysis, OXPHOS and fatty acid metabolism [18]. In the present study, we detected the local energy metabolic status and its prognostic value in patients with glioma with RNA sequencing data. Since energy metabolic gene could distinguish patients’ clinical and molecular features, we further developed a signature that could stratify patients with high or low-risk of poor outcome. Considering that univariate Cox model is insufficient for variables selection with dimensional data, we first performed univariate Cox model to filter genes related to OS and applied an elastic net regression Cox model to increase the predictive performance of the prognostic index [19], and the obtained 29 genes showed a cumulative effect on survival prediction. This energy metabolism-related signature could serve as a powerful prognostic indicator and stratify patients for energy metabolism-targeted therapies in future.

Functional analysis suggested that differences of biologic processes between high-risk and low-risk groups of patients were mainly involved in immune and inflammatory response, indicating an interface between energy metabolism and immune environment. Recently, compelling studies have identified numerous alterations in glioma cells metabolism that may play an important role in immune regulation [20]. The accumulation of lactic acid from aerobic glycolysis in tumor cells can shape the immune system, including increasing the transcription of cytokines, inhibiting differentiation of monocytes to dendritic cells [21,22]. Expression of *IDO1* (indoleamine 2, 3-dioxygenase 1), tryptophan metabolic enzyme, increases the recruitment of regulatory T cells and negatively impacts survival in glioma cells [23]. *IDO1* inhibition combined with *PD-L1* and *CTLA-4* inhibitors can enhance the therapeutic efficacy [24]. M2 macrophages use arginine to produce ornithine and urea, leading to anti-inflammatory effects and CD4+ T cell-mediated immune suppression [25]. To further understand the relationship between this risk score and immune response, immune checkpoints (*PD-1*, *PD-L1*, *CTLA-4*, *CD80* and *TIM-3*) [26–28] and inflammatory genes (*INF-α*, *INF-γ*, *TNF-α*, *IL-6*, *IL-17*, *CCL2*, *CXCL2 and HLA-A*) [29–32] were selected. Correlation analysis revealed that expression of these immune checkpoints was positively correlated with the risk score in both TCGA and CGGA cohorts (Supplementary Figure 10A and B), indicating an immunosuppressive state in high-risk group of glioma patients. In addition, the risk score was also positively associated with the expression of *INF-γ*, *IL-6*, *CCL2* and *HLA-A* (Supplementary Figure 10C and D), suggesting that macrophages and T cell mediated immune response were involved in high-risk group of glioma patients.

Collectively, we uncovered the energy metabolism gene expression and its prognostic value in diffuse glioma and identified an energy metabolism-related signature which could classify glioma patients with high-risk and low-risk groups of reduced survival. However, more prospective studies were further needed and the predictive ability of this signature should be tested for clinical application. Our findings offer new understanding about energy metabolism status and will benefit energy metabolism-targeted therapies in glioma.

## MATERIALS AND METHODS

### Datasets

The TCGA RNA sequencing data and corresponding clinical information, such as age, gender, histology, methylguanine methyltransferase (MGMT) promoter status, isocitrate dehydrogenase (IDH) mutation status and survival information, were downloaded from TCGA database (http://cancergemome.nih.gov/) as training set. Similarly, the CGGA RNA sequencing data and clinical information ware downloaded from CGGA database (http://www.cgga.org.cn) as validation set [33]. The characteristics of glioma patients from these two datasets were listed in Table 4.

**Table 4:** Clinical characteristics of diffuse glioma patients.

**TCGA cohort (550)**			**CGGA cohort (309)**	
**Characteristic**	**No.**		**Characteristic**	**No.**
**Age**			**Age**	
≤48	287		≤43	166
>48	263		>43	143
**Gender**			**Gender**	
Male	319		Male	194
Female	231		Female	115
**Subtype**			**Subtype**	
Classical	141		Classical	69
Mesenchymal	31		Mesenchymal	65
Proneural	345		Proneural	99
Neural	33		Neural	76
**Grade**			**Grade**	
II	191		II	104
III	211		III	67
IV	148		IV	138
**IDH**			**IDH**	
Mut	338		Mut	155
WT	212		WT	154
**MGMT promoter**			**MGMT promoter**	
Methylated	383		Methylated	136
Unmethylated	135		Unmethylated	111
NA	32		NA	62

IDH = isocitrate dehydrogenase; MGMT = methylguanine methyltransferase.

### Consensus clustering

Two energy metabolism-related gene sets (Reactome energy metabolism and energy-requiring part of metabolism) were downloaded from Molecular Signature Database v5.1 (MSigDB) (http://www.broad.mit.edu/gsea/msigdb/) [34]. Overlapped genes were removed and the acquired energy metabolism-related gene set contained 587 genes. Measured by median absolute deviation (MAD), the most variable genes were used for subsequent clustering. Consensus clustering was performed with R package “ConsensusClusterPlus”. The optimal number of subgroups was evaluated using cumulative distribution function (CDF) and consensus matrices [35].

### Gene signature identification

Significance analysis of microarray (SAM) was performed to identify the differentially expressed energy metabolism-related genes between lower grade glioma (LGG) and GBM with R package “samr”. Simultaneously, univariate Cox analysis was used to determine the prognosis-related genes. After that, the Cox proportional hazards model was applied for selection of optimal prognostic gene set with R package “glmnet”, which was suitable for the regression analysis of high-dimensional data [19]. Risk score for each patient of the TCGA training set was calculated with the linear combinational of the signature gene expression weighted by their regression coefficients. Risk score = (expr_gene1_ x coefficient_gene1_) + (expr_gene2_ x coefficient_gene2_) + … + (expr_genen_ x coefficient_genen_). Then, the regression coefficients from the training set was applied into the CGGA validation set for risk score calculation.

### Gene ontology (GO), gene set enrichment analysis (GSEA) and principal components analysis (PCA)

GO analysis was applied for the main function annotation of differential expression genes (http://david.ncifcrf.gov/). GSEA was performed to identify gene sets of statistical difference between two groups by using the GSEA v3 software (http://www.broadinstitute.org/gsea/index.jsp) [34]. PCA was carried out to detect expression difference within groups using R package “princomp” [36].

### Statistical analysis

According to the risk score, patients were divided into high-risk and low-risk groups based on the median value. Kaplan-Meier with 2-sided log-rank test was used to evaluate the overall survival (OS) differences between these two groups. Chi-square test was performed to detect the difference of the pathologic features between these two groups of patients. Univariate and multivariate Cox regression analysis was conducted to identify independent prognostic factors. ROC curve analysis was used to predict OS with R package “pROC”. All statistical analyses were conducted using SPSS or R software. *P*<0.05 was considered significant.

## SUPPLEMENTARY MATERIAL

Supplementary Table 1

Supplementary Table 2

Supplementary Table 3

Supplementary Figure 1

Supplementary Figure 2

Supplementary Figure 3

Supplementary Figure 4

Supplementary Figure 5

Supplementary Figure 6

Supplementary Figure 7

Supplementary Figure 8

Supplementary Figure 9

Supplementary Figure 10
