# Supplementary material for: Identification of an energy metabolism-related signature associated with clinical prognosis in diffuse glioma
Source: Aging (Albany NY). 2018 Nov 8;10(11):3185–209. doi: 10.18632/aging.101625 (PMC6286858; doi:10.18632/aging.101625)
Supplement: Supplementary Table 1 [file aging-10-101625-s001.pdf]

Supplementary Table 1. Characteristics of patients in class 1 and class 2 in CGGA cohort.

Characteristics	n	Class 1	Class 2	*P*-value
**Total Cases**	309	185	124	
**Age**				
≤43	166	80	86	**<0.001**
>43	143	105	38	
**Gender**				
Male	194	120	74	0.401
Female	115	65	50	
**Subtype**				
Classical	69	64	5	**<0.001**
Mesenchymal	65	65	0	
Proneural	99	44	55	
Neural	76	12	64	
**Grade**				
II	104	17	87	**<0.001**
III	67	41	26	
IV	138	127	11	
**IDH**				
Mut	155	51	104	**<0.001**
WT	154	134	20	
**MGMT promoter**				
Methylated	136	79	57	**<0.001**
Unmethylated	111	81	30	
NA	62	25	37	

IDH = isocitrate dehydrogenase; MGMT = methylguanine methyltransferase.
