# Supplementary material for: Identification of an energy metabolism-related signature associated with clinical prognosis in diffuse glioma
Source: Aging (Albany NY). 2018 Nov 8;10(11):3185–209. doi: 10.18632/aging.101625 (PMC6286858; doi:10.18632/aging.101625)
Supplement: Supplementary Table 2 [file aging-10-101625-s002.pdf]

Supplementary Table 2. Univariate and multivariate Cox regression analysis of clinical pathologic features for OS in CGGA cohort.

Characteristics	Univariate analysis			Multivariate analysis		
	HR	95% CI	*P*-value	HR	95% CI	*P*-value
**Age**	1.038	1.022-1.053	**<0.001**	0.999	0.983-1.016	0.914
**Gender**	0.843	0.597-1.189	0.33			
**Grade**	3.469	2.709-4.443	**<0.001**	2.097	1.511-2.91	**<0.001**
**Subtype**	0.583	0.492-0.691	**<0.001**	0.782	0.659-0.929	0.005
**IDH**	0.257	0.179-0.37	**<0.001**	1.106	0.566-2.159	0.768
**MGMT Promoter**	0.529	0.374-0.75	**<0.001**	0.78	0.53-1.147	0.207
**Risk score**	2.232	1.912-2.607	**<0.001**	1.556	1.161-2.086	**0.003**

HR = hazard ratio; CI = confidence interval; IDH = isocitrate dehydrogenase; MGMT = methylguanine methyltransferase.
