# Supplementary material for: Identification of an energy metabolism-related signature associated with clinical prognosis in diffuse glioma
Source: Aging (Albany NY). 2018 Nov 8;10(11):3185–209. doi: 10.18632/aging.101625 (PMC6286858; doi:10.18632/aging.101625)
Supplement: Supplementary Table 3 [file aging-10-101625-s003.pdf]

Supplementary Table 3. Characteristics of patients in low-risk and high-risk groups in CGGA cohort.

Characteristics	n	Risk score		*P*-value
		Low	High	
**Total Cases**	309	154	155	
**Age**				
≤43	166	112	54	**<0.001**
>43	143	42	101	
**Gender**				
Male	194	91	103	0.181
Female	115	63	52	
**Subtype**				
Classical	69	8	61	**<0.001**
Mesenchymal	65	3	62	
Proneural	99	86	13	
Neural	76	57	19	
**Grade**				
II	104	92	12	**<0.001**
III	67	36	31	
IV	138	26	112	
**IDH**				
Mut	155	136	19	**<0.001**
WT	154	18	136	
**MGMT promoter**				
Methylated	136	79	57	**<0.001**
Unmethylated	111	30	81	
NA	62	45	17	

IDH = isocitrate dehydrogenase; MGMT = methylguanine methyltransferase.
