# Supplementary material for: Identification of an energy metabolism-related signature associated with clinical prognosis in diffuse glioma
Source: Aging (Albany NY). 2018 Nov 8;10(11):3185–209. doi: 10.18632/aging.101625 (PMC6286858; doi:10.18632/aging.101625)
Supplement: Supplementary Figure 1 [file aging-10-101625-s004.pdf]

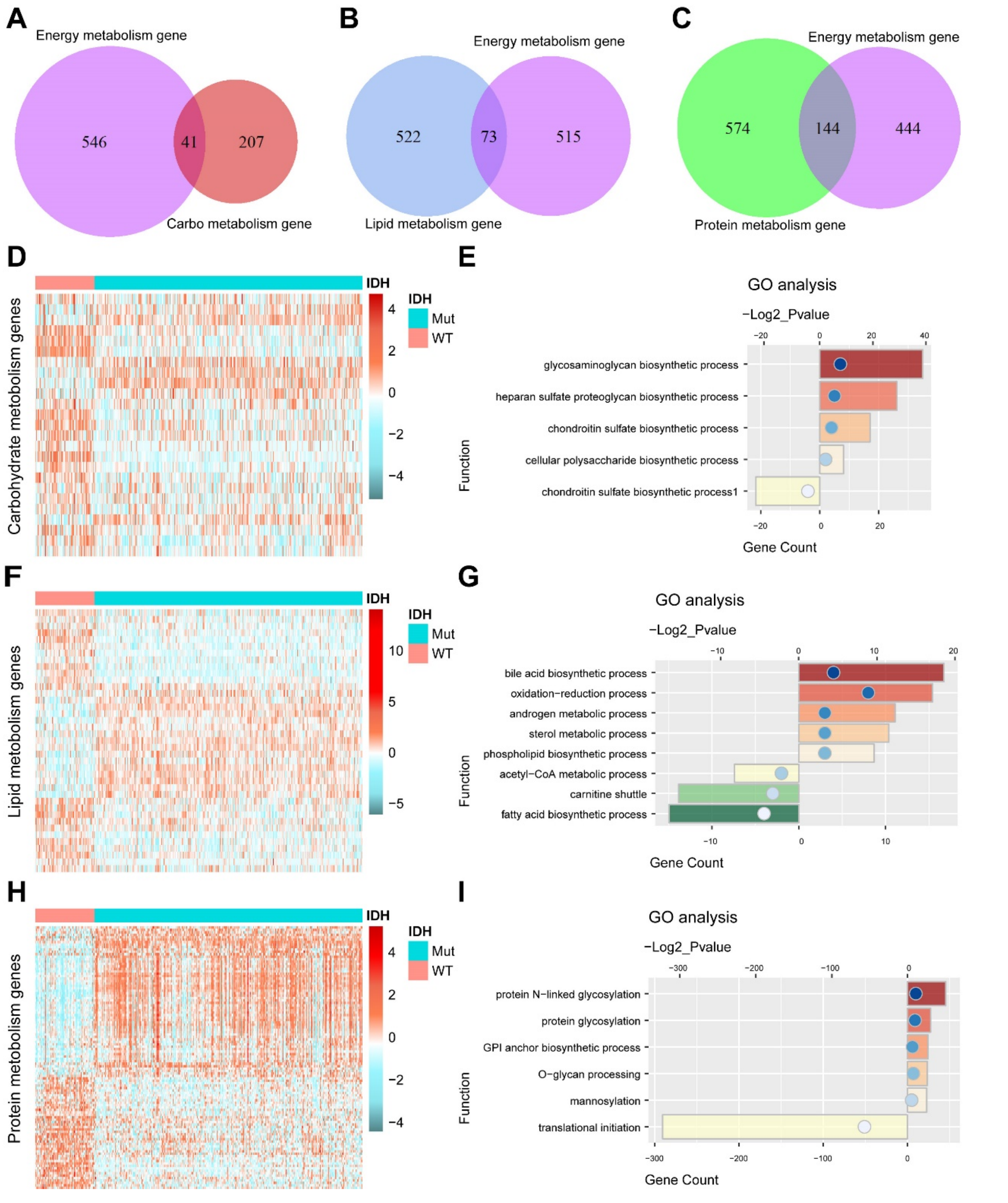

Supplementary Figure 1. Profile of carbohydrate, lipid and protein metabolism genes involved in energy metabolism between IDH-wt and IDH-mut LGG. (**A-C**) Venn diagrams show carbohydrate, lipid and protein metabolism genes involved in energy metabolism. (**D** and **E**) Heat map and GO analysis of differentially expressed carbohydrate metabolism genes between IDH-wt and IDH-mut LGG. (**F** and **G**) Heat map and GO analysis of differentially expressed lipid metabolism genes. (H and I) Heat map and GO analysis of differentially expressed protein metabolism genes.
