# Supplementary material for: Identification of an energy metabolism-related signature associated with clinical prognosis in diffuse glioma
Source: Aging (Albany NY). 2018 Nov 8;10(11):3185–209. doi: 10.18632/aging.101625 (PMC6286858; doi:10.18632/aging.101625)
Supplement: Supplementary Figure 2 [file aging-10-101625-s005.pdf]

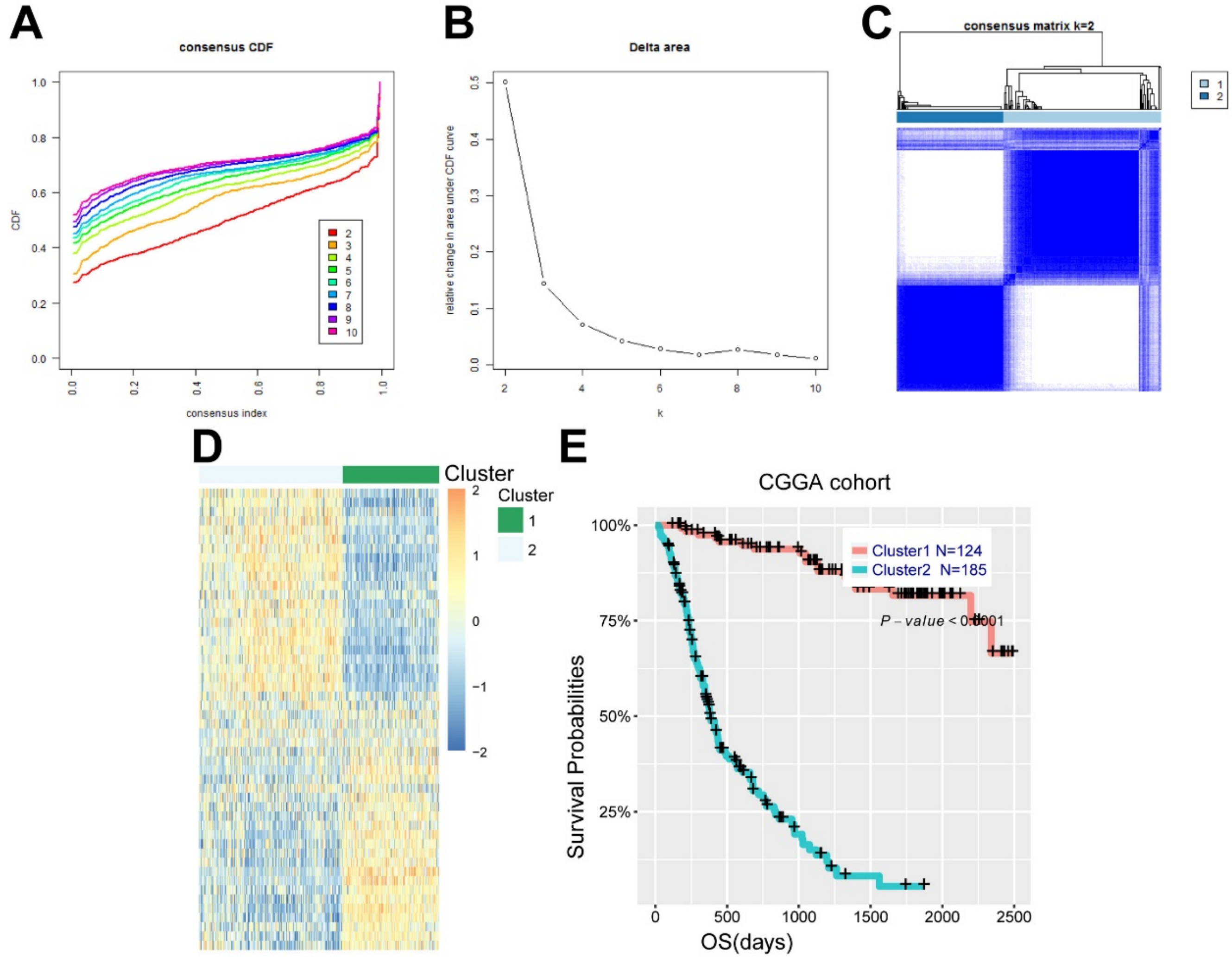

Supplementary Figure 2. Energy metabolism-related genes could distinguish glioma patients with different clinical and molecular features. (**A**) Consensus clustering CDF for k = 2 to k = 10. (**B**) Relative change in area under CDF curve for k = 2 to k = 10. (**C**) Consensus clustering matrix of 309 samples from CGGA dataset for k = 2. (**D**) Heat map of two clusters defined by the top 50 variable expression genes. (**E**) survival analysis of patients in cluster 1 and cluster 2.
