# Supplementary figures and images for: Identification of an energy metabolism-related signature associated with clinical prognosis in diffuse glioma
Source: Aging (Albany NY). 2018 Nov 8;10(11):3185–209. doi: 10.18632/aging.101625 (PMC6286858; doi:10.18632/aging.101625)

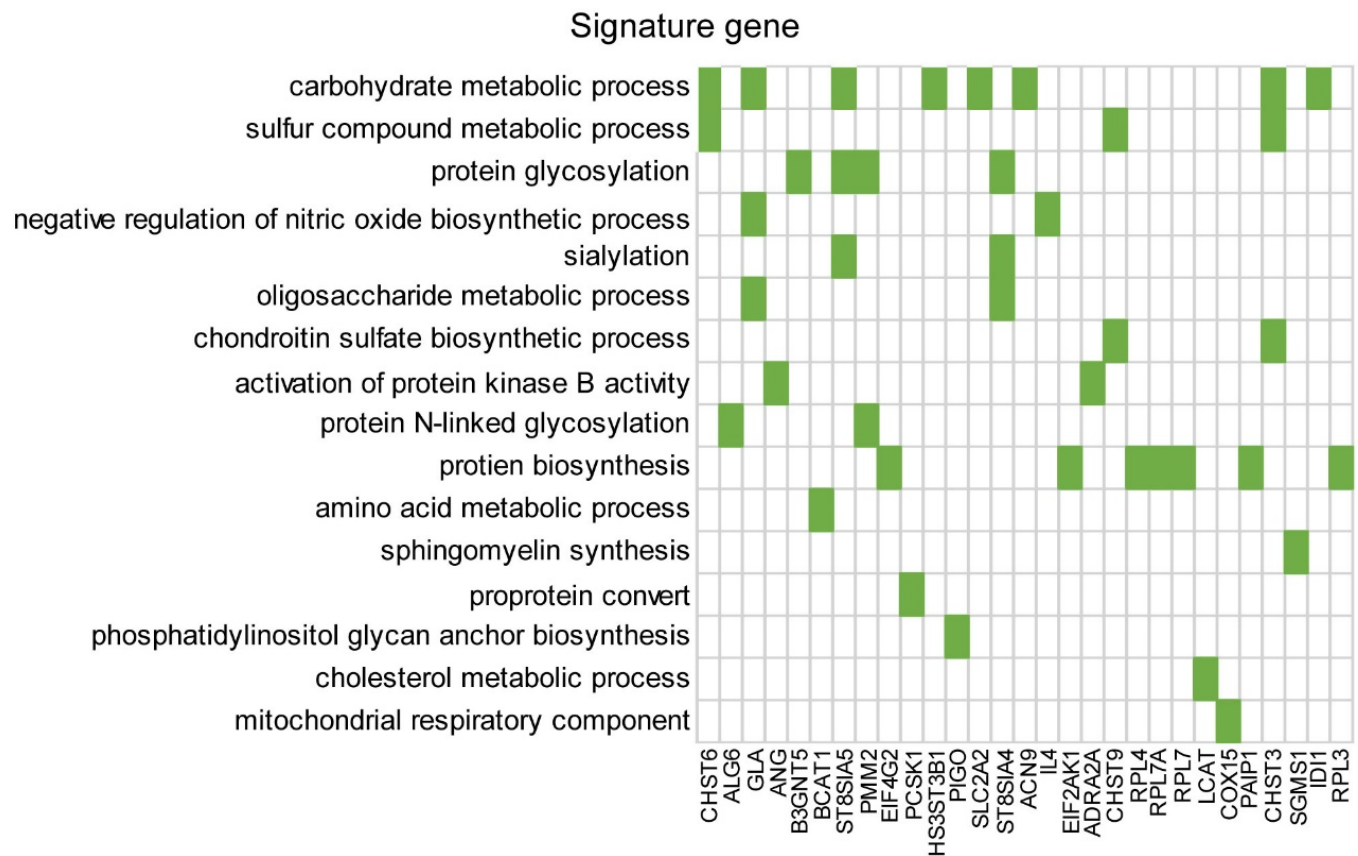

**Supplementary Figure 3. Functional annotation of the signature genes.**

Supplement: Supplementary Figure 3 [file aging-10-101625-s006.pdf]
