# Supplementary material for: Identification of an energy metabolism-related signature associated with clinical prognosis in diffuse glioma
Source: Aging (Albany NY). 2018 Nov 8;10(11):3185–209. doi: 10.18632/aging.101625 (PMC6286858; doi:10.18632/aging.101625)
Supplement: Supplementary Figure 4 [file aging-10-101625-s007.pdf]

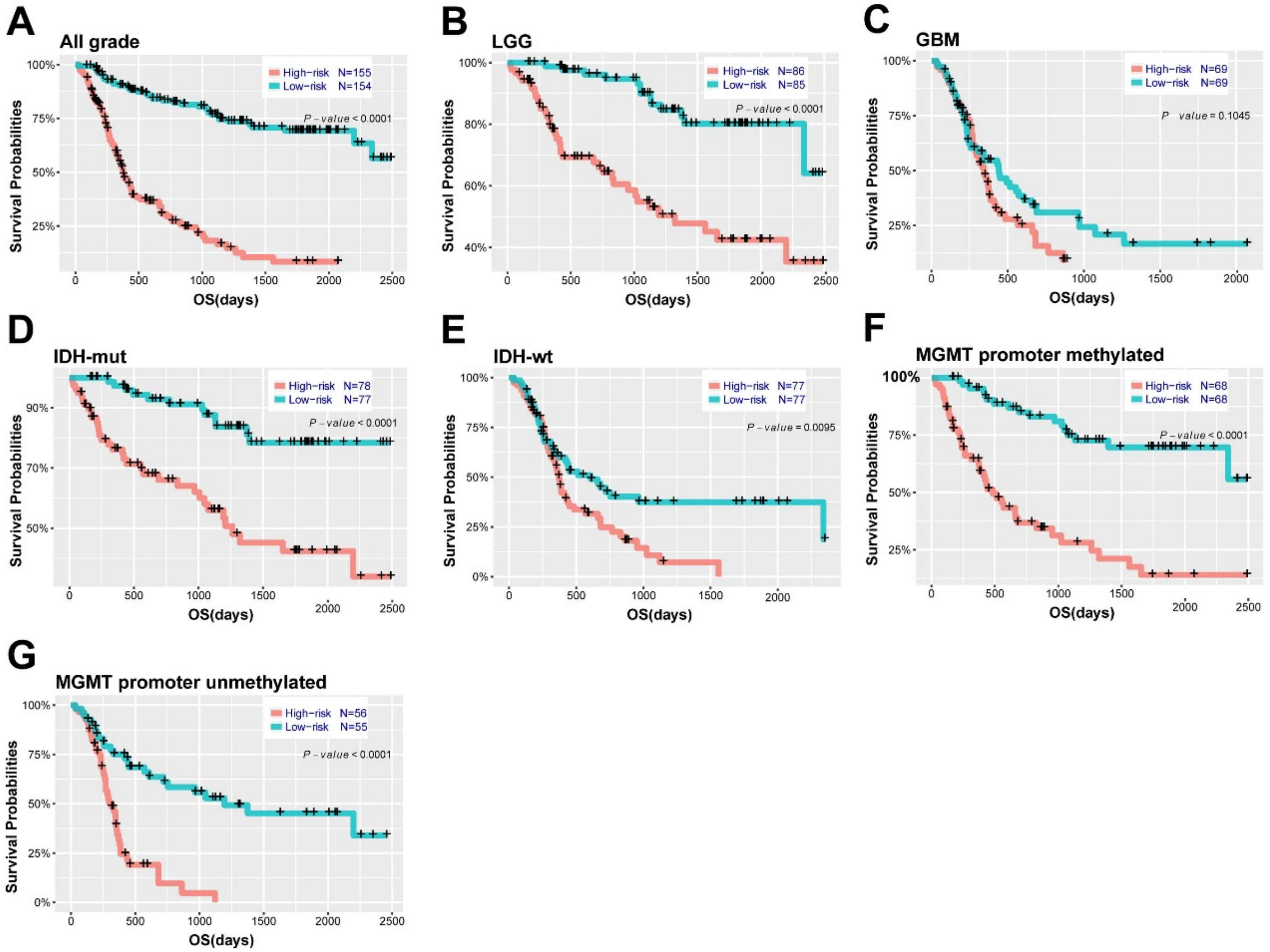

Supplementary Figure 4. Prognostic evaluation of the 29-gene signature in stratified patients of CGGA cohort. (**A-G**) Survival analysis of the signature in patients stratified by grade, IDH and MGMT promoter status.
