# Supplementary material for: Identification of an energy metabolism-related signature associated with clinical prognosis in diffuse glioma
Source: Aging (Albany NY). 2018 Nov 8;10(11):3185–209. doi: 10.18632/aging.101625 (PMC6286858; doi:10.18632/aging.101625)
Supplement: Supplementary Figure 5 [file aging-10-101625-s008.pdf]

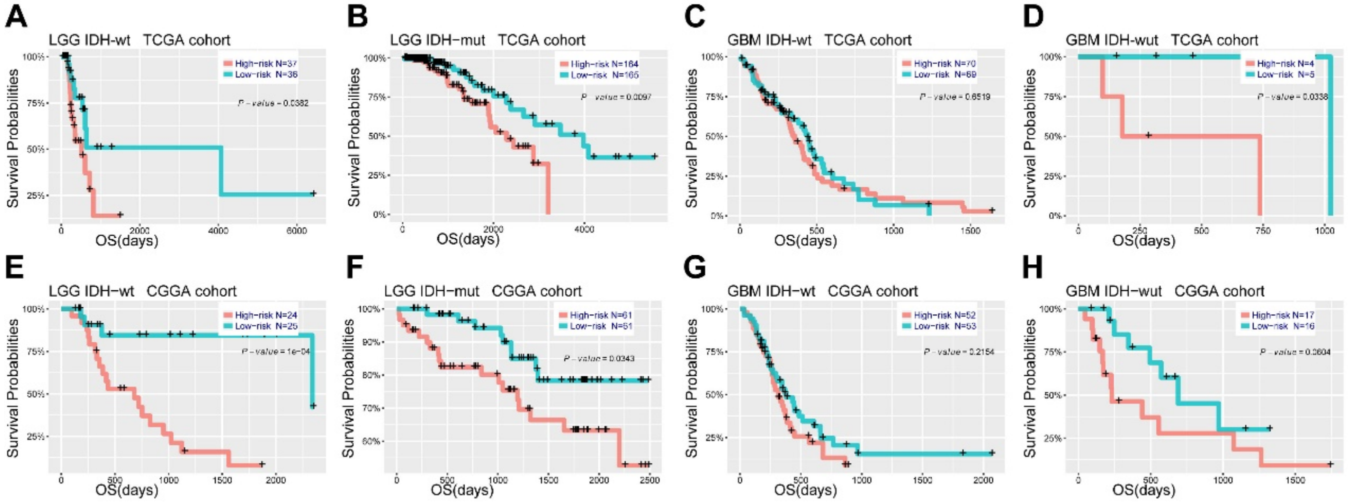

Supplementary Figure 5. Prognostic evaluation of the 29-gene signature in molecular subgroups. (**A-D**) Survival analysis of the signature in LGG IDH-wt, LGG IDH-mut, GBM IDH-wt and GBM IDH-mut patients of TCGA cohort. (**E-H**) Survival analysis of the signature in LGG IDH-wt, LGG IDH-mut, GBM IDH-wt and GBM IDH-mut patients of CGGA cohort.
