# Supplementary material for: Identification of an energy metabolism-related signature associated with clinical prognosis in diffuse glioma
Source: Aging (Albany NY). 2018 Nov 8;10(11):3185–209. doi: 10.18632/aging.101625 (PMC6286858; doi:10.18632/aging.101625)
Supplement: Supplementary Figure 6 [file aging-10-101625-s009.pdf]

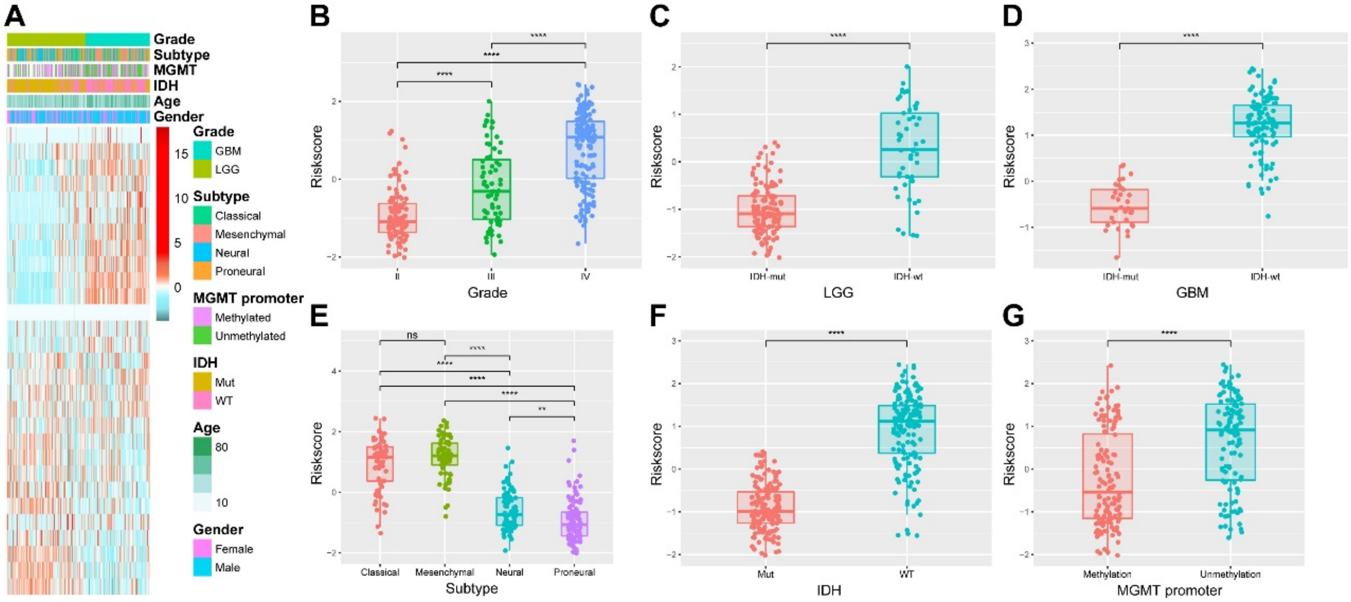

Supplementary Figure 6. Association between the energy metabolism-related signature and clinical features in CGGA cohort. (**A**) Heatmap of the 29 genes of the signature based on the risk score value. (**B-G**) Distribution of the risk score in stratified patients by grade, subtype, IDH and MGMT promoter status.
