# Supplementary material for: Identification of an energy metabolism-related signature associated with clinical prognosis in diffuse glioma
Source: Aging (Albany NY). 2018 Nov 8;10(11):3185–209. doi: 10.18632/aging.101625 (PMC6286858; doi:10.18632/aging.101625)
Supplement: Supplementary Figure 7 [file aging-10-101625-s010.pdf]

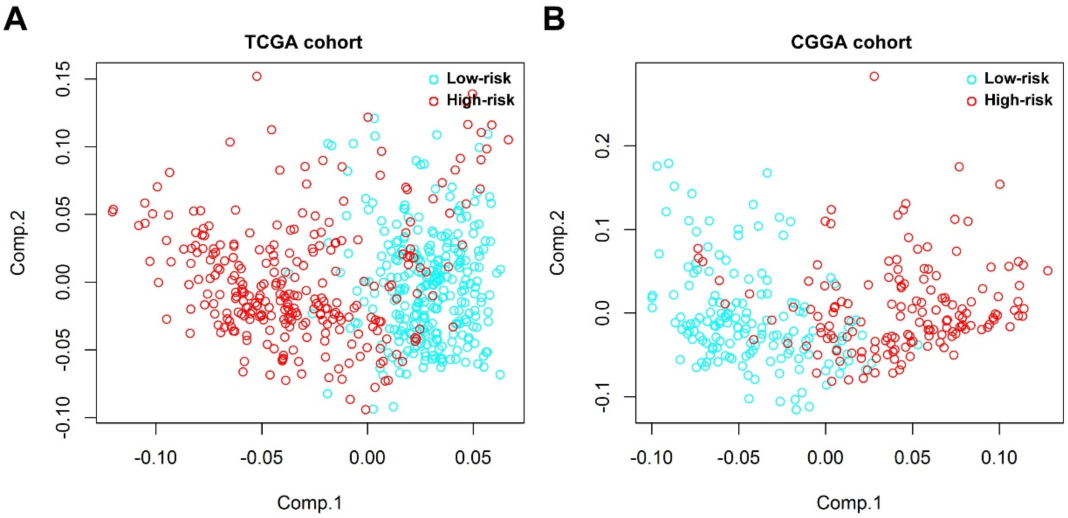

Supplementary Figure 7. Principal components analysis of high and low-risk groups of patients based on whole gene expression data. (**A-B**) PCA in TCGA and CGGA cohorts.
