# Supplementary material for: Identification of an energy metabolism-related signature associated with clinical prognosis in diffuse glioma
Source: Aging (Albany NY). 2018 Nov 8;10(11):3185–209. doi: 10.18632/aging.101625 (PMC6286858; doi:10.18632/aging.101625)
Supplement: Supplementary Figure 8 [file aging-10-101625-s011.pdf]

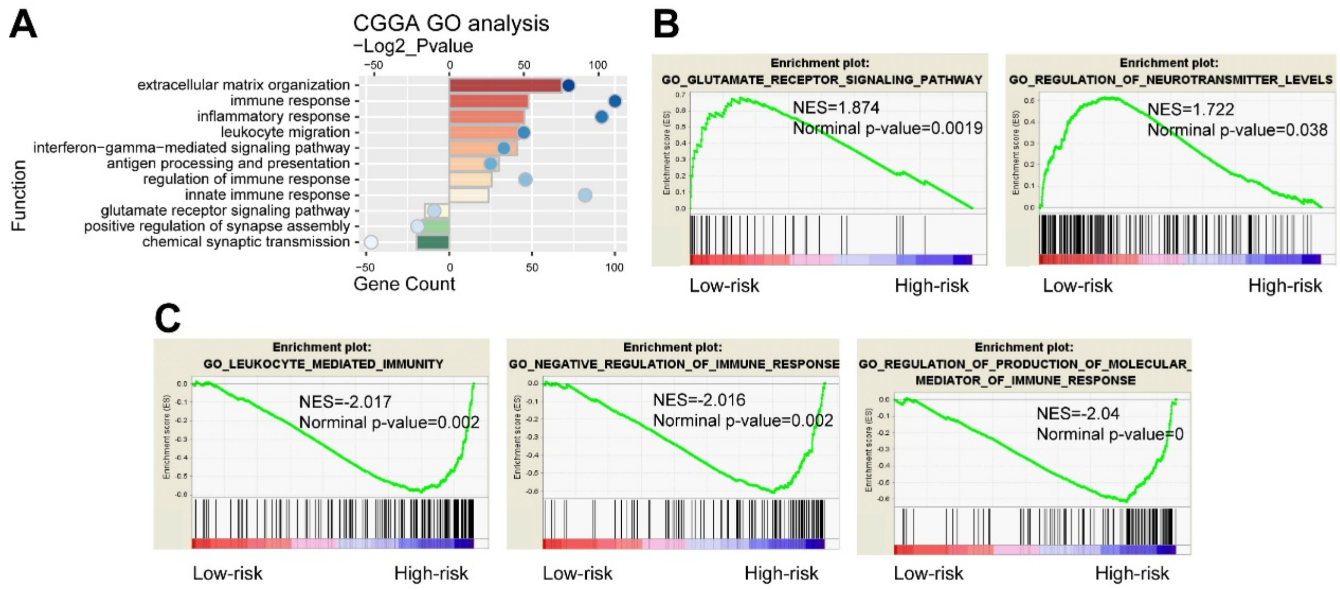

Supplementary Figure 8. Functional analysis of the 29-gene signature in CGGA cohort. (**A**) GO annotations based on the top 2000 genes positively and negatively associated with the 29-gene signature. (**B**-**C**) GSEA analysis based on the median value of risk score.
