# Supplementary material for: Identification of an energy metabolism-related signature associated with clinical prognosis in diffuse glioma
Source: Aging (Albany NY). 2018 Nov 8;10(11):3185–209. doi: 10.18632/aging.101625 (PMC6286858; doi:10.18632/aging.101625)
Supplement: Supplementary Figure 9 [file aging-10-101625-s012.pdf]

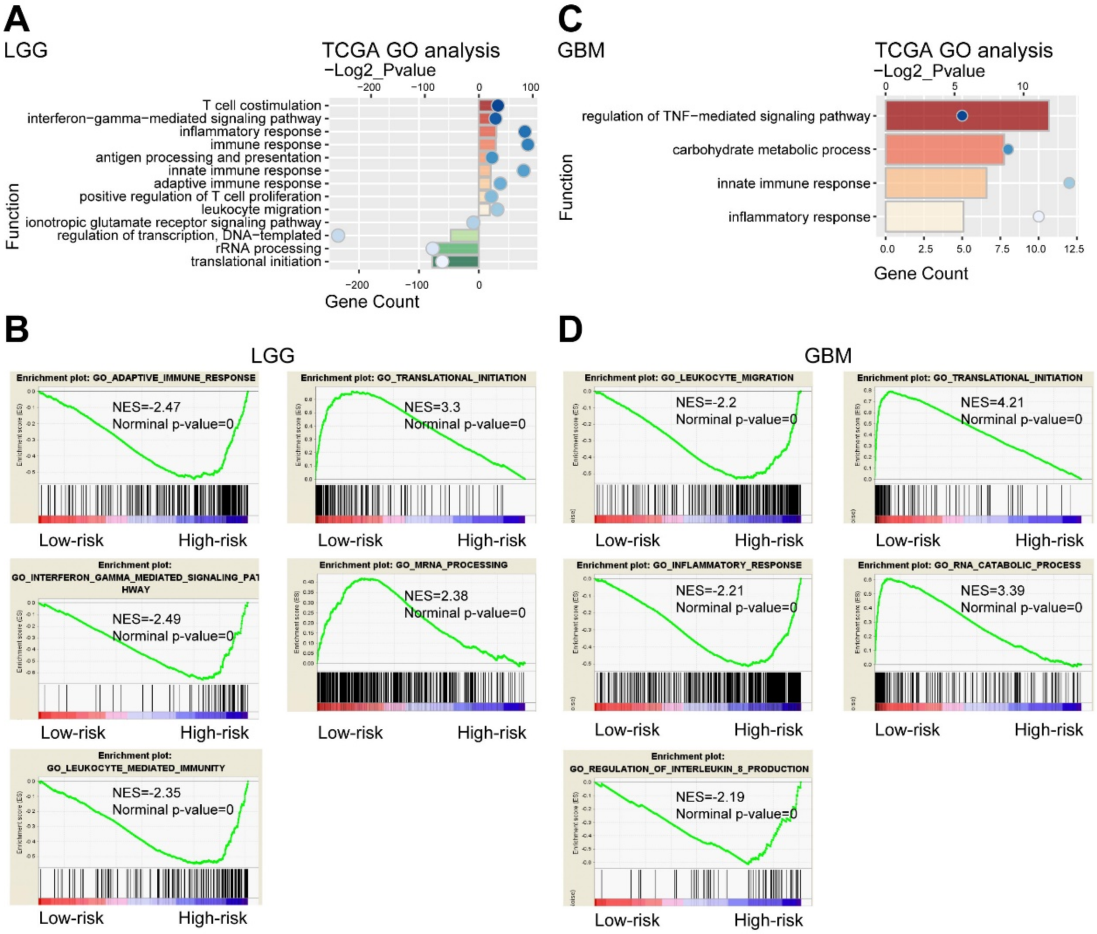

Supplementary Figure 9. Functional analysis of the 29-gene signature in LGG and GBM of TCGA cohort. (**A**) GO annotations based on the top 2000 genes positively and negatively associated with the 29-gene signature in LGG. (**B**) GSEA analysis based on the median value of risk score in LGG. (**C**) GO annotations based on the top 2000 genes positively and negatively associated with the 29-gene signature in GBM. (**D**) GSEA analysis based on the median value of risk score in GBM.
