# Supplementary material for: Identification of an energy metabolism-related signature associated with clinical prognosis in diffuse glioma
Source: Aging (Albany NY). 2018 Nov 8;10(11):3185–209. doi: 10.18632/aging.101625 (PMC6286858; doi:10.18632/aging.101625)
Supplement: Supplementary Figure 10 [file aging-10-101625-s013.pdf]

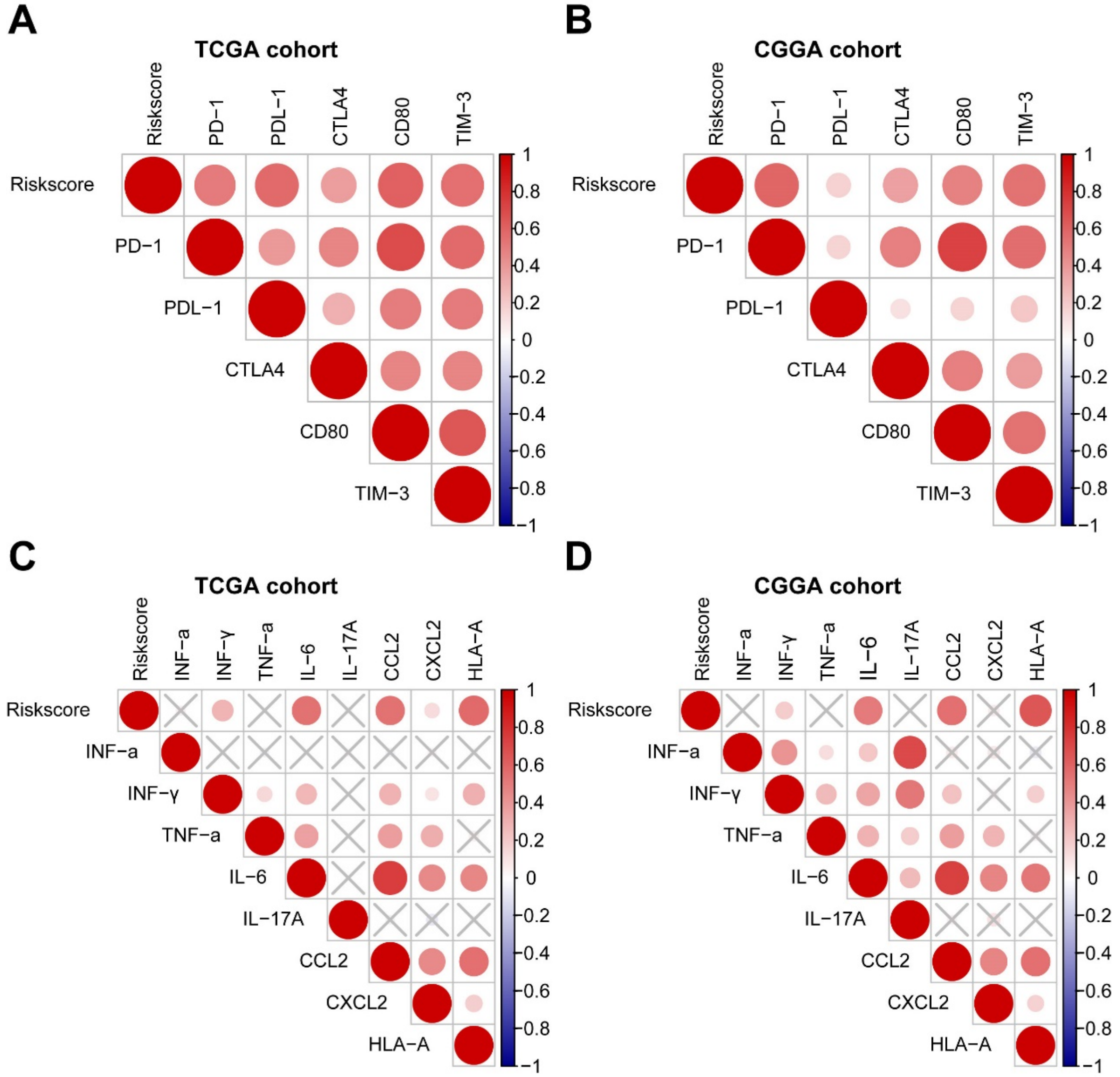

Supplementary Figure 10. Association between the energy metabolism-related signature and immune, inflammatory responses. (**A-B**) Correlation analysis between risk score and immune checkpoints. (**C-D**) Correlation analysis between risk score and inflammatory genes.
